# Can 3D imaging modeling recognize functional tissue and predict liver failure? A retrospective study based on 3D modelling of the major hepatectomies after hepatic modulation

**DOI:** 10.1186/s12893-023-02196-z

**Published:** 2023-10-18

**Authors:** Emilio Vicente, Yolanda Quijano, Hipolito Duran, Eduardo Diaz, Isabel Fabra, Luis Malave, Pablo Ruiz, Giada Pizzuti, Chiara Naldini, Giovanni De Nobili, Riccardo Caruso, Valentina Ferri

**Affiliations:** 1 Division of General Surgery, Sanchinarro Hospital, San Pablo University, Calle Oñaa 10, 28050 Madrid, Spain; 2 Università Degli Studi Di Pavia, Pavia, Italy; 3 Università Degli Studi Di Padova, Padua, Italy; 4 Università Degli Studi Gabriele d’Annunzio Chieti Pescara, Pescara, Italy

**Keywords:** Mayor hepatectomy, Post operative  liver  failure, 3d  reconstruction, Functional imaging

## Abstract

**Background:**

Thanks to the introduction of radiomics, 3d reconstruction can be able to analyse tissues and recognise true hypertrophy from non-functioning tissue in patients treated with major hepatectomies with hepatic modulation.The aim of this study is to evaluate the performance of 3D Imaging Modelling in predict liver failure.

**Methods:**

Patients submitted to major hepatectomies after hepatic modulation at Sanchinarro University Hospital from May 2015 to October 2019 were analysed.

Three-dimensional reconstruction was realised before and after surgical treatment. The volumetry of Future Liver Remnant was calculated, distinguishing in Functional Future Liver Remnant (FRFx) i.e. true hypertrophy tissue and Anatomic Future Liver Remnant (FRL) i.e. hypertrophy plus no functional tissue (oedema/congestion) These volumes were analysed in patients with and without post hepatic liver failure.

**Results:**

Twenty-four procedures were realised (11 ALPPS and 13 PVE followed by major hepatectomy). Post hepatic liver failure grade B and C occurred in 6 patients. The ROC curve showed a better AUC for FRFxV (74%) with respect to FRLV (54%) in prediction PHLF > B. The increase of anatomical FRL (iFRL) was superior in the ALPPS group (120%) with respect to the PVE group (73%) (*p* = 0,041), while the increase of functional FRFX (iFRFx) was 35% in the ALLPS group and 46% in the PVE group (*p* > 0,05), showing no difference in the two groups.

**Conclusion:**

The 3D reconstruction model can allow optimal surgical planning, and through the use of specific algorithms, can contribute to differential functioning liver parenchyma of the FLR.

**Supplementary Information:**

The online version contains supplementary material available at 10.1186/s12893-023-02196-z.

## Introduction

Complete surgical resection is the gold standard treatment with potential curative intent for patients with hepatocellular carcinoma (HCC), intrahepatic cholangiocarcinoma (CI), or metastatic disease [1]. However, curative surgical resection remains challenging not only in terms of achieving a negative margin but also in maintaining sufficient future liver remnant (FLR) to avoid post-hepatectomy liver failure (PHLF) [2]. Associated liver partition and portal vein ligation (ALPPS) and portal vein embolization (PVE) are the most commonly used techniques used for FRL regeneration. The functional increase of the FRL does not always correspond to the volumetric increase, therefore, a functional study of the future remnant is essential before completing the hepatectomy.

In the last decade, 3D diagnostic models have been gaining acceptance in the hepatic field allowing an increased understanding of liver anatomy and a more accurate planning of surgery [3]. Although companies as MEVIS or CellaMS have already proven that 3D modelling technology is a field that helps and give support to clinical profiles providing key aspects of interest for surgery planning as the identification of portal territories, the emerging field of radiomics seems to be an additional tool to add value to these models including functional information based on medical images while reducing the invasiveness for the patient. The new frontier of these diagnostic models is represented by the fact that they could also distinguish between hypertrophic and functioning regenerated liver, adding information on the functional state of the liver and thus helping in predicting patients with PHLF.

The present study aims to quantify liver hypertrophy using the 3D Cella Medical Solutions (3D-MSP®) imaging technique to distinguish between anatomical future remnant liver (FRL) and functional future remanent liver (FRFx) patients treated with major hepatectomy after hepatic regeneration techniques.

## Materials and methods

This is a retrospective study carried out at Sanchinarro University Hospital, Madrid from May 2015 to October 2019, analysing patients with insufficient FLR volume that required modulation before major hepatectomy. Associated liver partition and portal vein ligation (ALPPS) and portal vein embolisation (PVE) followed by major hepatectomy were the technique used.

For each patient, a 3D imaging reconstruction (3D-MSP®) was performed before and after the liver regeneration technique and the data of anatomical and function FLR volumetry were analysed.

Sanchinarro University Hospital is a reference centre in hepatobiliary surgery with extensive experience in major hepatectomies and especially in the ALPPS technique, which has been described in previous reports without open and robotic approach [4, 5].

### Patients selection

Inclusion criteria were patients with unilateral or bilateral liver malignancies with a FLR to total liver volume (TLV) ratio (FLR/TLV) < 25% and FLR/TLV < 40% when liver parenchyma damage (steatosis, fibrosis or chemotherapy-induced liver damage) was suspected in the radiological and laboratory tests. We included patients with Child A liver function and patent right portal vein.

Functional assessement of patients candidate to surgery consisted on assessment of the degree of ascites and evaluation of the presence and severity of encephalopathy. Bioquemical assessment including the levels of bilirubin and albumin and the prothrombin time. Patients were stratified according to the Child Pugh classification.

Exclusion criteria were signs of portal hypertension, such as ascites, and/or intra-abdominal varices, the presence of distant metastasis, and complete right portal vein thrombosis.

The preoperative study consisted of tumour markers (CEA, CA19-9), computed tomography (CT) scans, magnetic resonance imaging (MRI) and positron emission computed tomography (PET TC) and/or positron emission magnetic resonance imaging (PET MRI).

PHLF was defined as the postoperative deterioration of liver function with an increase in the INR and concomitant hyperbilirubinemia on or after postoperative day 5, as proposed by the International Study Group of Liver Surgery (ISGLS) [6]. Postoperative complications were recorded and categorized according to the modified Clavien-Dindo classification [7].

### Acquisition and processing of 3D Cella Medical Solutions (3D-MSP®) imaging

A 3D Cella Medical Solutions (3D-MSP®) imaging reconstruction was created for each patient after liver regeneration techniques. In patients undergoing ALPPS, this was performed preoperatively, and one week before the second surgical procedure. In patients undergoing hepatic embolisation, this was performed preoperatively, and one week before the scheduled hepatectomy.

#### 3D Imaging acquisition methods

##### Data acquisition

all available preoperative images of the patient (CT, MRI, PET-CT, PET-MRI) are used to analyze tumour distribution, estimate remaining liver volume, and identify tumour-vessel relationships with vascular anatomy. An image acquisition protocol is used to normalize the characteristics of the acquired images (3dMSP) (Video 1). The data are obtained in DICOM format (Digital Imaging and Communication in Medicine) and are anonymized.

In this study, CT images only were used in order to prevent the incorporation and fusion of other methods from altering the volumetric results.

##### Image fusion

The different modelled elements are demarcated in the most appropriate diagnostic imaging sequences. Therefore, the use of image fusion techniques is necessary to correct errors resulting from breathing, movement, position, etc., in the patient. Rigid registration techniques are used for image rotation, translation and scaling alignment, while similar, non-rigid registration techniques are used for 3D imaging for tissue deformation correction.

##### Image pre-processing and segmentation

Hepatic parenchyma, CVI, supra-hepatic vein, portal vein, hepatic artery, bile duct, and tumour are segmented. If necessary, other structures, such as cysts, hilar adenopathies, prostheses, staples, drainages, etc., are also reconstructed. Noise is previously reduced with anisotropic diffusion filters and N3 algorithms.Convolutional networks and advanced medical image processing techniques as active contour and region growth are used for segmentation.

##### Processing of modelling

Laplacian smoothing filters are used in the 3D reconstruction of the model to correct the scaling derived from the thickness of the scan. In addition, techniques are used to divide the parenchyma into hepatic segments I-VIII and to subdivide the vascular elements: supra-hepatic in LHV, MHV and RHV; port in RPV, LPV and MPV; artery in LHA, RHA, CHA-Aorta; bile ducts in the gallbladder, cystic, common bile duct, common liver and bile ducts. The models are processed to allow the execution of regulated and unregulated resections, virtual ablations and safety margins of 5, 10 and 15 mm, obtaining the remaining and tumour volumes. Finally, algorithms were developed for the identification of portal and arterial anatomical variants based on atlases and other 3D pattern recognition.

#### Liver function analysis

Once the imaging studies have been analysed according to inclusion criteria, the segmentation of the structures of interest for analysis is conducted. In this case, the liver parenchyma, arterial and venous vasculature, portal vasculature, tumour regions, and gallbladder are segmented. With the aim of avoiding the impact of vascular and tumour tissues on the acquired textures, radiomic feature extraction of the radiomic characteristics of the liver parenchyma is performed exclusively, removing the vascular and tumour regions from the analysis. These annotations of the structures of interest was performed in both pre-diagnosis CT images and pre-surgery CT images.

Thus, considering ALPSS and embolisation patients as a whole group, radiomics features were extracted on FLR tissue and analysed blindly in all patients. In this way, the goal was to reach the characteristics that could be associated algorithmically to find similarities among all pre-diagnosis studies (considering the parenchyma as functional tissue) and differences with hypertrophied tissue. This provided the features that seemed to be indicators of the differentiation between functional and oedema tissue after hypertrophy on pre-surgery images.

**Fig. 1:**
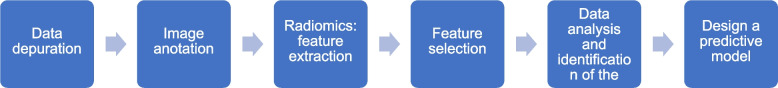
Workflow of radiomics algorithm

The workflow of the algorithm is showed in Fig. 1.

The software to extract and analyse the features was developed in-house based on PyRadiomics library, validated by the IBSI imaging biomarker standardisation initiative (current reference standard for the development of radiomic studies. A total of 106 first order (histogram/intensity-based), shape, and second and higher order (texture-based) features are extracted for each of the imaging studies selected in the analysis. A standardisation of the extracted characteristics is conducted.

Due to the high number of extracted characteristics, it is necessary to conduct a selection of those that contain relevant information for the analysis. In this preliminary study, the information gain metric is used to select the characteristics of first order that add greater value to the analysis. The values of the selected characteristics are analysed through box plots. Additionally, the Student t-test statistical test (assuming normal distributions) was used to compare the mean values of each radiomic characteristic for both study groups. Additionally, an unsupervised clustering method (k-Means) is evaluated for the identification of possible subgroups of studies based on the activation profiles of the radiomic characteristics. Finally, the performance of a multivariate logistic regression model based on selected radiomic characteristics for the classification of liver functional volume above or below the defined threshold based on the texture profile of the liver parenchyma is evaluated.

**Table:** 

General requirements for the acquisition of CT images	
Acquisition window	All anatomical structures of interest should be visualized
Scan resolution	Distance between scans ≤ thickness (contiguous scans). Distance between scans: max 30% overlap
Image size / Pixel size	- Constant image and pixel size in each sequence - Minimum size of the matrix 512 × 512
Gantry inclination	Without inclination
Breathing phase	It must be the same in all explorations
Other Recommendations	- Avoid motion artifacts and achieve light, relaxed breathing - Avoid unnecessary metal artifacts - Acceptable image disturbance - Volume of intravenous contrast medium that provides a correct visualization of the structure of interest
Archive format	DICOM

**Table:** 

General requirements for MR imaging	
Acquisition window	All anatomical structures of interest should be visualized
Cut resolution	- Distance between cuts ≤ cut thickness (cuts contiguous) - Distance between cuts ≤ 6 mm
Image Size / Pixel Size	- Constant image and pixel size within each sequence - Minimum size of the Matrix 512 × 512
Other recommendations	- Gentle breaths without sudden movements - Uniform signal on all anatomical structures - Tolerable image noise - For tumor localization, an examination protocol is required that produces a sufficiently high contrast for this. It is advisable that you always include a fat suppression sequence (STIR, SPAIR, or similar) - For images of vascular anatomy, the acquisition of dynamic images with contrast in 2 or 3 times is recommended
Archive format	DICOM

**Table:** 

Specific requirements for MR imaging in 3D-MSP Hepatic Surgery	
- **Dynamic sequences**	- Cutting distance: ≤ 6 mm - Acquisition of dynamic images in 2 times arterial and venous) or in 3 times (arterial, venous, late venous) - Use bolus tracking
- **Fat suppression sequences**	- Cutting distance: ≤ 6 mm - For the localization of tumors - STIR, SPAIR or similar
- **MRI cholangiography**	- Cutting distance: ≤ 6 mm - Sections oriented axially or coronally (axially recommended)

### 3D liver volume analysis

The following volumetric were calculated in the 3D model:

- TV, the tumour volume
- The total liver volume (TLV) was calculated before starting the regeneration procedure
- The Anatomic Future Remnant Liver (FRL: hypertrophy + oedema) was calculated by manual delineation according to Couinaud’s functional segmentation of the liver [8]. FRL is the anatomic volumetric value, it includes true hypertrophy tissue + edema/congestion
- The Functional Future Liver Remanent (FRFx: real hypertrophy) is the real hypertrophy, functional tissue and it was calculated with version 1.2.1 and was used for the first-order liver structure analysis of the algorithm for quantifying and oedema-congestion.

The volume of the FRL (FRLV, %) and the FRFx (FRFxV, %) was calculated using the following formula:

$$\mathrm{FRLV}(\mathrm{\%}) =\mathrm{ FRL }/ (\mathrm{TLV}-\mathrm{TV})\times 100$$

$$\mathrm{FRFxV}(\mathrm{\%}) =\mathrm{ FRFx }/ (\mathrm{TLV}-\mathrm{TV})\times 100$$

The increase of the FRL and FRFx were calculated using the following formula:

$$\mathrm{iFRL\%}= (\mathrm{FRLpost}-\mathrm{FRL})/\mathrm{FRL x }100$$

$$\mathrm{iFRFx\%}= (\mathrm{FRFxpost}-\mathrm{FRFx})/\mathrm{FRFx x }100$$

Additionally, for each patients we considered:

- The standardised FRL (sFRL) that was calculated using a formula based on BSA [9]:
  $$\mathrm{sFLR}=\mathrm{ FRL}/-794.41 +\mathrm{ 1,267.28 xBSA}$$

The BSA was calculated with the following formula:

$$\mathrm{BSA}= \surd \mathrm{ height}(\mathrm{cm})\mathrm{ x weight}(\mathrm{kg})/3600$$

- The kinetic growth rate (KGR) was defined as the percentage-point difference between the liver volume and remnant liver before and after the intervention or surgery and it was calculated as percentage growth per day [10].

### Outcome measures

Primary endpoint was to correlate the volumetric values with clinical outcomes, analysing the Anatomic Future Remnant Liver (FRL) and the the Functional Future Liver Remanent (FRFx) in patients with PHLF B,C and in patients with PHLF A.

Secondary end point was correlate Anatomic Future Remnant Liver (FRL) and the The Functional Future Liver Remanent (FRFx) in patients treated with ALLPS and with PVE followed by mayor hepatectomy.

### Statistical analysis

Continuous variables reported were the median with interquartile range and categorical variables as frequency and absolute percentages. The normality criteria were tested on the cohort according to the Shapiro–Wilk test. The variables are compared with the Mann Whitney U test and chi-square for quantitative and qualitative data, respectively. Correlation between PHLF and post hepatic volume values were analysed with Spearman correlation.

For the statistical analysis, SPSS software (version 10, IBM SPSS, Chicago, IL, USA) was used and all tests were considered statistically significant with a value of *p* ≤ 0.05.

## Results

Our study included 24 patients treated with major hepatectomies after hepatic modulation. PHLF occurred in 10 (41%) patients as follows: grade A in four patients (17%), grade B in four patients (17%), and grade C in two patients (8%). Thirty-day post operative mortality occurred in 2 patients (8.3%) due to irreversible PHLF. Patients’ characteristics are shown in Table 1.

**Table 1:** Demographic, operative and postoperative characteristics according to post hepatic liver failure (PHLF)

	**PHLF < B** ***n* = 18**	**PHLF > B** ***n* = 6**	***p***
Age, years Median (range)	70 (42–77)	72 (60–79)	0,773
Sex n(%) M/F	12 (66)/6(33)	1 (17)/5(83)	0,048
BMI kg/m2 Median (range)	23,3 (21–27)	22 (19–24)	0,786
ASA n(%)			0,454
I-II	10 (56)	38 (50)	
III	8 (44)	3 (50)	
Bilirubine md/dl Median (range)	0,9 (0,14–3,72)	7,5 (4,57–14)	0,003
INR Median (range)	1,2 (1,05–1,57)	1,57 (1,20–2,5)	0,020
Creatinine mg/dl Median (range)	0,63 (0,31–1,22)	1,12 (0,75–1,77)	0,010
Histology type n (%)			0,321
IHCC	5 (27)	2 (33)	
Hepatocarcinoma	1 (6)	1 (16)	
Metastases	12 (67)	2 (33)	
Sclerosing Cholangitis	0	1 (16)	
Hepatectomy n (%)			0,127
Extended RH	11 (61)	2 (33)	
Extended LH	2 (11)	0	
RH	3 (17)	2 (33)	
RH + metastasect	2 (11)	0	
Extended RH + metastasectomy	2 (11)	1 (16)	
Extended LH + metastasectomy	0	1 (16)	
Chemotherapy n (%)			0,422
yes	11 (64,7)	3 (42,8)	
no	6 (35,3)	4 (57,2)	
Blood loss, ml Median (range)	500 (200–800)	200 (0–500)	0,655
Hospital stay, days Median (range)	28,7 (15–90)	9,2 (6–35)	0,272
Surgical Technique n (%)			0,560
PVE	3	10	
ALLPS	3	8	

### Primary outcome: liver hypertrophy analysis through 3D Cella Medical Solutions imaging (3D-MSP®) to predict PHLF

When analysing patients according to PHLF severity, we found that the functional future liver remnant volume (FRFxV: real hypertrophy) was inferior in the group of patients with PHLF ≥ B with respect to patients with PHLF < B (50% vs 34%, although without reaching statistical significance *p* = 0.096).

On the contrary there was not a difference in the anatomical future liver remnant volume (FRLV%: hypertrophy + oedema) value that resulted in 70% of patients with PHLF < B and in 72% of patients with PHLF ≥ B. In support of this result, sFRL also appears to be lower in patients with PHLF ≥ B (74% vs 62%) despite there is no statistically significant association.

**Fig. 2:**
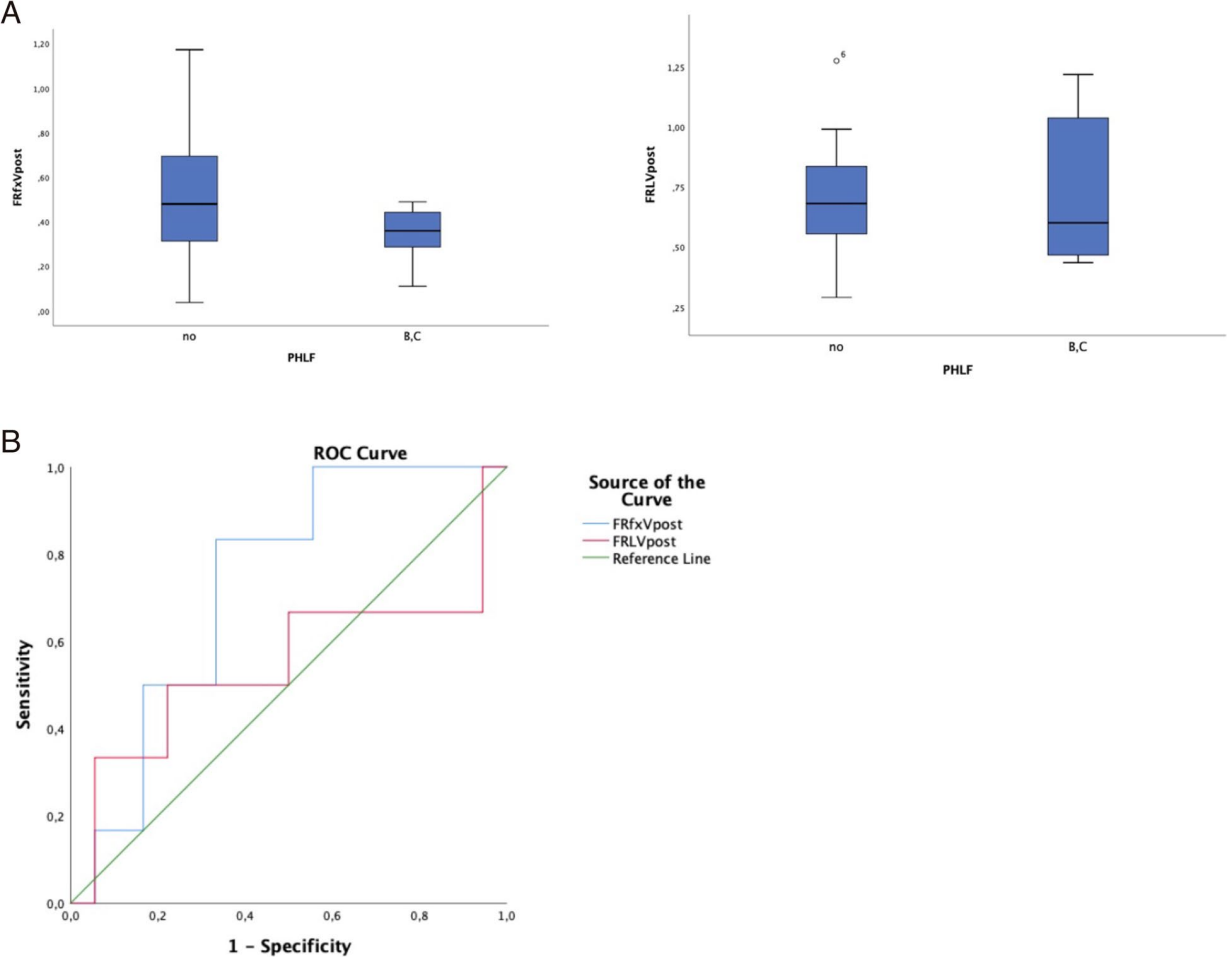
**A** FRLVpost and FRfxVpost values in patient with and without PHLF > B. **B** ROC Curve por FRLVand FLFxV, sFRL and KGR in predicting PHLF > B

In contrast, KGR resulted 2,8% in the ALPPS group and 0,04 in the PVE group, but do not provide significant value able to predict liver failure (Table 2). This analysis suggests that 3D imaging modelling is able to detect real hypertrophy in the hepatic parenchyma, in fact, patients with superior FRFxV value do not present major PHLF. Using the FLFxV and FLRV the ROC curve was constructed. The ROC curve for FLFxV was superior with respect to the FLRV, sFRL and KGR values with an area under the curve (AUC) of 0.731 (see Fig. 2).

**Table 2:** Posthepatectomy liver failure (PHLF)

Post values	PHLF < B *n* = 18	PHLF > B *n* = 6	*p*
FLR (cc) (Median, range)	1023,48 (662–1631)	941 (536–1651)	0,542
FLFx(cc) (Median, range)	672 (124–1502)	456 (128–761)	0.096
FLRV(%) (Median, range)	70 (29–127)	72 (44–122)	0,864
FLFxV(%) (Median, range)	50 (4–117)	34 (11–49)	0,096
iFLR(%) (Median, range)	93 (15–242)	101 (26–145)	0,763
iFLFx(%) (Median, range)	45 (-79–166)	29 (-71–146)	0,683
sFLR(%) (Median, range)	74 (53–117)	62 (39–99)	0,196
KGR (%/day) (Median, range)	1,54 (0–5)	1,51 (0–4)	0,997

### Secondary outcome: liver hypertrophy analysis through 3D Cella Medical Solutions imaging (3D-MSP®).according to surgical technique

The ALPPS technique was performed in 11 patients and 13 patients treated with PVE were selected (Table 3). Volumetric data are summarised in Table 4.

**Table 3:** Demografic according to surgical technique

	**ALPPS** ***n* = 11**	**PVE** ***n* = 13**	***p***
Age, years (Median, range)	70,5 (61–67)	67 (42–81)	0,567
Sex (M/F) n(%)	7 (63,6)/4(36,4)	6(46,2)/7(53,8)	0,444
ASA n(%)			0,754
I	1(9)	2(16)	
II	7(63,6)	8(61)	
III	3(27,3)	3 (23)	
BMI	24.1 (21,1–29)	26.4 (22,3–28,1)	0,456
Lesion number n(%)	3,2(1–10)	2,1 (1–7)	0,150
Tumor size mm (bigger lesion) (Median, range)	72 (15–120)	59 (35–180)	0,287
Number of hepatic segment interest n(%)	3 (2–6)	2 (1–6)	0,232
Neoadjuvant chemotheraty n(%)			
yes	7(63,6)	7(53,8)	0,855
no	4 (36,4)	6(46,2)	
Enlapse time between stage /PVE and mayor hepatectomy. Days (Median, range)	13,9 (7–22)	66 (36–106)	< 0,01
KGR (%/day)	2,8	0,4	< 0,01
Hepatectomy n(%)			0,101
Extended RH	6(54)	7(54)	
Extended LH	1(9)	1(8)	
RH	0	5(38)	
RH + metastasect	2(18)	0	
Extended RH + metastasectomy	1(9)	0	
Extended LH + metastasectomy	1(9)	0	
Associated biliary resection n (%)			
yes	2(18,2)	4(30)	0,356
no	9(81,8)	9(70)	
Caval resection			0,213
no	11(100)	11(84)	
Resection	0	1(8)	
Trombectomy	0	1(8)	
Histology type n(%)			
IHCC	3(27,3)	4(30)	0,318
Hepatocarcinoma	0	2(16)	
Metastasis	8(72,7)	6(46)	
Other	0	1(8)	
Intraoperative trasfusione, ml (Median, range)	450 (0–600)	300 (0–800)	0,564
Hospital stay, days (median, range)	22 (6–90)	7,9 (6–35)	0,101
Dindo Clavien > III n (%)			
yes	2(18)	4(30)	0,431
no	9(72)	9(70)	
PHLF			0,989
no	6(55)	8(61)	
A	2(18)	2(16)	
B	2(18)	2(16)	
c	1(9)	1(8)	
30 Day Mortality			0,692
yes	1(9)	1(8)	
no	10(91)	12(92)	
90 Day Mortality			0,222
yes	2(22)	1(8)	
no	9(78)	12(92)	

*RH* right hepatectomy, *LH* left hepatectomy, *PHLF* post hepatic liver failure

**Table 4:** Volumetric analysis between ALPPS and PVE group

Volumetric and functional parameters	ALPPS	PVE	*p*
**Baseline, median [IQR]**			
** TLVpre (cc)**	1318 (1051–3894)	1480 (1051–3894)	0,111
** FRLpre (cc) (Median, range)**	350 (240–839)	649 (297–1023)	**0,040**
** FRFxpre(cc) (Median, range)**	312 (215–733)	579 (264–898)	**0,040**
** FRLVpre (%) (Median, range)**	29 (19–61)	34 (20–68)	0,259
** FRFxVpre (%) (Median, range)**	26 (16–54)	29 (18–60)	0,245
**Post-PVE or pre stage 2, median [IQR]**			
** Time (days) between stage 1 and CT**			
** TLVpost (cc) (Median, range)**	1681 (1412–2177)	1884 (1381- 4241)	0,212
** FRLpost (cc) (Median, range)**	901 (535–1651)	1055 (711–1631)	0,339
** FRFxpost (cc) (Median, range)**	417 (124–1171)	810 (128–1502)	**0,049**
** FRLVpost (%) (Median, range)**	71 (43–121)	63 (29–127)	0,748
** FRFxVpost(%) (Median, range)**	31 (13–77)	48 (3–117)	0,168
** iFLR (%) (Median, range)**	120,98	**73,85**	**0,041**
** iFRFx (%) (Median, range)**	35,47	**46, 47**	**0,743**

The preoperative FRL value was inferior in the ALPPS group (350 cc, range 240 cc-839 cc) with respect to the PVE group (649 cc, range 297 cc-1023 cc). In addition, the preoperative FxFR was inferior in the ALPPS group (312 cc, range 215 cc-733 cc) with respect to the PVE group 579 cc (264 cc-898 cc) (*p* = 0,040).

**Fig. 3:**
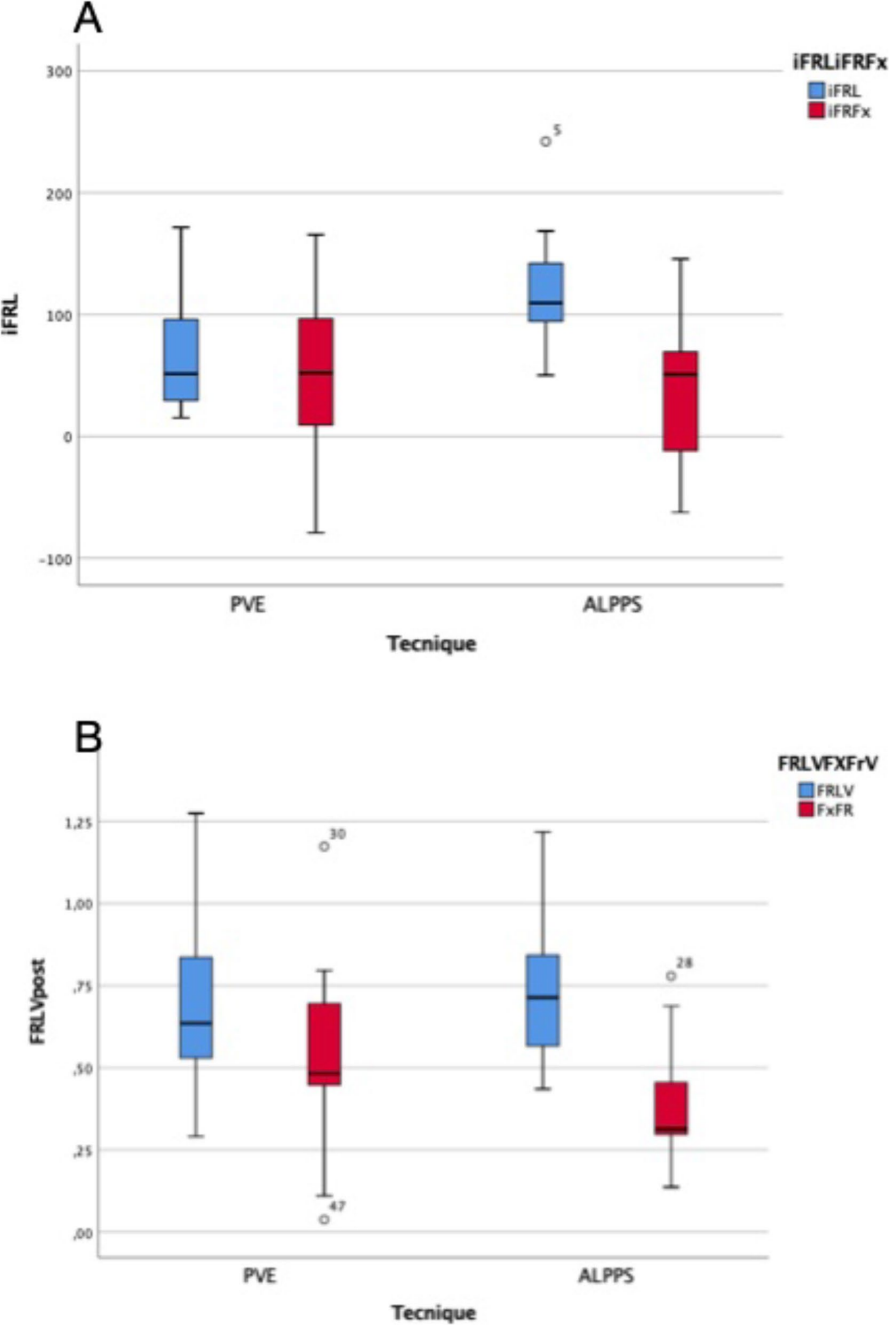
**A** Anatomical future liver remanent increase (iFRL) and Functional future liver increase (iFRFx) in PVE and ALPPS group. **B** Anatomic future liver remanent /total liver volume (FLRV) and Functional future liver remanent /total liver volume (FLFxV) after hepatic regeneration process in PVE and ALPPS groups

Regarding the post-regeneration values, the increase of anatomical future liver remnant (iFRL) was 73.85% in the PVE group and 120.98% in the ALPPS group, showing a statistically significant increase (*p* = 0,04) in patients submitted to ALLPS. Moreover, the increase of functional liver remnant (iFLFX) was similar in the two groups, with a value of 46.47 in the PVE group and 35,54 in the ALPPS group (*p* = 0,743) (see Fig. 3). This analysis demonstrated a lack of correspondence between anatomical and functional growth. In the ALPPS group, the anatomical future liver remnant/total liver volume (FLRVpost%) was 71(43–121) and the functional future liver remnant/total liver volume (FRFxVpost%) was 31(13–77) (*p* = 0,02). In the PVE group, FLRVpost% was 63(29–127) and FRFxVpost% results 48(3–117) (*p* = 0,141) (see Fig. 1B). Differences between FRLVpost and FRFxpost were significantly superior in the ALPPS groups with respect to the PVE, supporting the fact that the ALPPS technique increases anatomical volume without a proportional functional increase.

## Discussion

Extended hepatectomy is a challenging surgical technique that can be planned in many hepatic disorders, such as metastatic disease, hepatocellular carcinoma and intrahepatic cholangiocarcinoma, where limitations are represented by the possible onset of hepatic failure [11, 12]. In order to avoid PHLF, a sufficient hepatic remnant had to be assured, and with this aim several vascular manipulations have been described in order to induce hypertrophy of the residual liver parenchyma.

The embolization of the portal vein (PVE) described by Makuuchi in 1980 and [13] and the associated liver partition and portal vein ligation (ALPPS) technique introduced by Shnitzbauer et al. in 2012 [14] are the most commonly used strategies to reach hypertrophy, however anatomical increase in the hepatic parenchyma does not always correspond to a functional increase.

In recent years, 3D modelling has gained acceptance in hepatic surgery, allowing better visualisation and comprehension of the internal anatomy of the liver [15, 16]. The application and digital intelligent diagnostic may provide an individualized display of the complex internal anatomy of the liver and the spatial information of liver and its surrounding organs. This enables surgeons to obtain relatively comprehensive information to assist in clinical decision-making [17]. 3D visualisation has great potential in displaying lesion information and guiding diagnosis and treatment. Zang et al. demonstrated in his meta-analysis that the application of 3D visualisation in primary hepatocellular carcinoma had an extremely significant impact in terms of the following: reduced intraoperative blood loss; postoperative complications; operating time and hospitalization time; recurrence rate of liver cancer in short-term follow-up; and significantly accelerating the recovery of postoperative liver function [18].

Furthermore, in major hepatectomies, 3D reconstructed models can visualise and intuitively display variations of intrahepatic blood vessels and provide a convenient and accurate method for liver volume calculation, virtual simulation surgery, and surgical navigation [19, 20]. Advances in technology have improved the 3D simulation software technology to allow the analysis of liver function.

In recent studies Lopez-Lopez et al. described a multicentre study of 35 patients, and they compared degree of similarity in vascular calibres and in the distances between the tumour and vessel between 3D printing and CT/ MRI. They conclude that 3DP hepatic models present a good correlation compared with CT/MRI and surgical pathology, and they are useful for education, understanding, and surgical planning, but does not necessarily affect the surgical outcome [21]. Another interesting study recently published [22] with a population of 136 patients with resectable hepatocellular carcinoma suggests that preoperative 3D imaging can associated with a better prediction of the performed surgical procedure for liver resections in HCC compared to conventional imaging techniques. Another important application of 3-D is the creation of augmented reality in the operating theatre [23].

Therefore 3d reconstruction is an excellent tool for planning hepatic surgery, but its superiority to classical imaging techniques has not been clearly demonstrated.

In our opinion only radiomics can revolutionise diagnostic imaging identifying pathophysiological features of the image. This work for the first time describes the real advantage of 3d imaging technology in liver surgery i.e. it can allow to quantify the functionality of the hepatocyte and thus of the remaining liver.

Moreover artificial intelligence and introduction of radiomic may allow the identification of hepatic functional panrenquima even in the preoperative setting and cold lead to quantify the preoperative hepatic damage induced by chemotherapy or cholestasis.

3D Cella Medical Solutions (3D-MSP®) has proposed an imaging analysis that can detect functioning hepatic parenchyma by distinguishing cellular hypertrophy versus oedema and congestion. An algorithm recognises different hepatic texture, allowing a distinction between anatomic future liver remnant (FRL, compose by real hypertrophy and oedema/congestion) and functional future liver remnant (FRFx that represents the real hypertrophy).

In this study postoperative clinical outcomes were analysed and compared with the anatomical and functional volumetry: PHLF grade B and C occurred in six patients: the value of functional FRFxV was inferior in patients with PHLF ≥ B, although it does not show statistical significance (*p* = 0.096), suggesting that FRFxV may be a predictive factor of PHLV. Furthermore, the Area Under the Curve (AUC) of the ROC curve showed a better performance in predicting PHLV B of FRFxV with respect to FLRV, sFRL and KGR. These are promising results that need to be validated in a larger population.

In the preoperative volume analysis, patients in the ALPPS group present a significantly inferior FRL and FRFx volume, while the patients in the PVE group support the fact that the ALPPS technique was indicated for those patients for which the possibility of performing a major hepatectomy was related to the need for an extra increase in the remaining future. The elapsed time between the regeneration technique and surgery was significantly superior in the PVE group, with respect to the ALPPS group (66 days vs 13 days, respectively).

Regarding the post regeneration liver value, we observed a statistically significant increase of anatomic FRL (iFRL) in the ALPPS group with respect to the PVE group, while the increase of functional FRFX (iFRFx) does not present a significant increasing. These data may support the lack of correspondence between the anatomical and functional increase in patients after ALPPS regeneration technique, as has been described in several studies [20]. This difference is due to the production of a certain amount of oedematous and congestive tissue during the hepatic regenerative process, mostly in the ALPPS group, where the regenerative process takes place in considerably reduced times. Functional studies, such as hepato-biliary-scintigraphy, have been employed to test hepatic function shown as CT scan volumetry and have overestimated the function of the FLR by up to 50% [24] after the ALPPS procedure.

This study suggests that the new frontier of imaging in hepatic surgery can be represented by a radiological tool that unites the advantages of 3D diagnostics modelling and the possibility of performing an analysis of the liver texture through dedicated algorithms to differentiate between functional tissue and congestion oedema.

This study has several limitations.

First, results are based on a small number of patients in which we observed a very high incidence of severe PHLF (grade B or C) and an enlargement of the study cohort (e.g. by organizing a multicenter study) might be useful to obtain numbers that can be coherent with the worldwide literature. Second a comparison with a functional imaging technique such as hepatic scintigraphy has not been performed. Despite these limitations, this preliminary result shows that 3D modelling may help to distinguish between anatomical and functional liver remnants, allowing a preoperative prediction of post hepatic liver failure. Certainly, these are promising preliminary results, which must be validated by future larger studies.

### Supplementary Information

**Additional file 1.**

## Data Availability

Because of the sensitive nature of the data collected for this study, requests to access the dataset from qualified researchers trained in human subject confidentiality protocols may be sent to Valentina Ferri at valenpeglio@gmail.com.The datasets used and/or analysed during the current study available from the corresponding author on reasonable request.
